# Special anatomy series. Imaging inner ear structures with high-frequency ultrasound: Application to physical rehabilitation space medicine

**DOI:** 10.1097/ph9.0000000000000026

**Published:** 2024-01-25

**Authors:** Jeffrey Strakowski, Han Zhang, Millard Reschke, Faye Y. Chiou-Tan

**Affiliations:** a Department of Physical Medicine and Rehabilitation, The Ohio State University, Columbus, OH; b Department of Physical Medicine and Rehabilitation, Ohio Health Riverside Methodist Hospital, Columbus, OH; c Department of Neurobiology and Anatomy, McGovern University of Texas Health Science Center Houston, Houston, TX; d Department of Neuroscience, NASA Johnson Space Center, Houston, TX; e H. Ben Taub Department of Physical Medicine and Rehabilitation, Baylor College of Medicine, Houston, Texas

**Keywords:** Gait disturbance, balance, neurovestibular, inner ear, anatomy, space medicine, ultrasound

## Abstract

**Objective:**

The objective of this paper is to document the feasibility of image acquisition, image optimization, and sonographic appearance of the exposed anatomic windows of cadaveric inner ear dissection for purposes of potential future clinical evaluation as part of the developing area of physical and rehabilitation space medicine.

**Methods:**

Cadaveric dissection of the inner ear was conducted with the goal of exposing areas relevant to vestibular balance. Middle and inner ear structures of 3 human cadavers were imaged with multiple broadband transducers, including emphasis with higher frequency transducers.

**Results:**

The images were best optimized with 17 MHz and 22 MHz small footprint transducers. High-frequency ultrasound (US) images of the semicircular canals, vestibular and facial nerves, and utricles with reflected otoliths (otoconia) were obtained and reported in this article. Detailed visualization of both the vestibular nerve and facial nerve was accomplished, including identification of fascicular architecture. In addition, US reflection from the otoliths contained within the utricle was identified with sufficient clarity to provide surface measurements. Bony acoustic landmarks of the middle ear bones were identified by scanning externally from the tympanic membrane, including the dynamic movement of the bones with manual manipulation.

**Conclusion:**

US visualization has the potential to be an effective imaging modality to monitor potential changes to the otolith's size throughout extended space flight. To our knowledge, no prior study has reported US images of human inner ear structures.

## Introduction

The increasing physical demands of longer-duration space travel on astronauts will require a multidisciplinary team of medical professionals, including physiatrists and scientists, to both diagnose and treat medical complications to ensure success. As the goals for accomplishing unprecedented space flight times develop, innovation for effective monitoring of both expected and potentially unexpected complications is needed. A significant concern is the potential for adaptive tissue changes resulting from extended periods of reduced gravity that could impair function upon flight completion. Early identification of such changes could lead to improvement in adjustments to in-flight strategy, as well as appropriate rehabilitation efforts, to minimize them. The rapid recent technological advances in imaging with ultrasound (US) make it a promising modality for in-flight monitoring.

### Inner ear concerns

**Figure 1:**
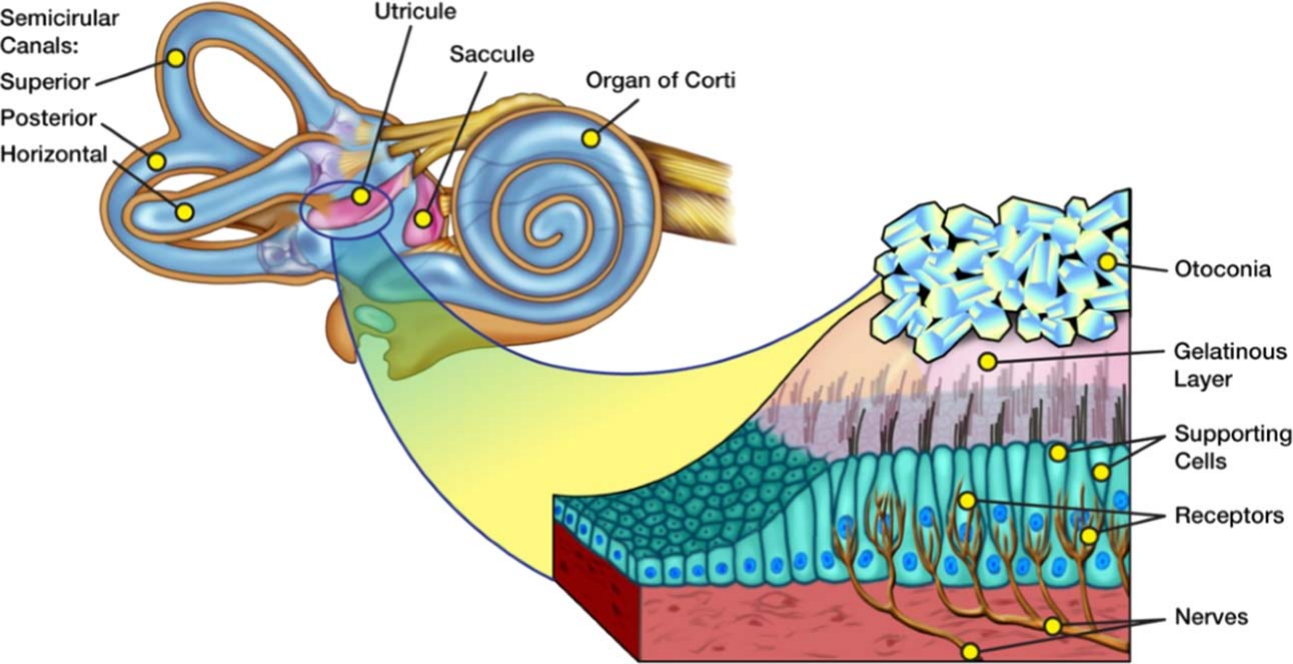
Illustration of the pertinent anatomy of the vestibular system in the inner ear. (Artwork by Cynthia Bush, NASA).

Diminished gravity during prolonged space travel has the potential to create adaptive changes to the vestibular system that could impair vestibular balance and equilibrium with a return to normal gravity. US monitoring of inner ear structures through extended space flight, such as to Mars, could help to identify alterations in the vestibular system and potentially lead to appropriate interventions. The objective of this paper is to determine the sonographic appearance of structures of interest in the inner ear in cadaveric specimens, as well as to determine the best transducer frequencies for image optimization. In this study, the middle and inner ear structures of 3 human cadavers were imaged with multiple broad-band transducers. Most of the image acquisition was obtained with 17 MHz and 22 MHz small footprint transducers. We describe the sonographic appearance of the semicircular canals, vestibular and facial nerves, and utricles with reflected otoliths (also known as otoconia, Fig. 1).

### Anatomic and ultrasound considerations

The inner ear is located in the petrous part of the temporal bone. It includes the cochlea, vestibule, and semicircular canal. Functions include sound reception and balance. Shearing forces acting upon the otoliths (both utricular and saccular) provided by linear accelerations and the Earth’s gravitational field together with the semicircular canals (angular acceleration) in the inner ear help create vestibular balance and equilibrium. Long-term dysfunction can occur in the human vestibular system from a prolonged reduction in gravity during extended space travel^1^. A prolonged weightless environment creates the potential for adaptive changes in the structure of otolithic stones, cilia formation, and both afferent and efferent neurons. Gradual changes will distort normal equilibrium once the astronauts return to a normal Earth’s gravity. Otoliths can be displaced, become misshapen, or enlarge without the usual sensory and gravitational input that is normally present on Earth’s 1G conditions.

On Earth, high-resolution computerized tomography (CT) is used for osseous labyrinth visualization and magnetic resonance imaging (MRI) for examining membranous and neural structures^2^. CT and MRI are used in a complementary manner to look at the bony structure and cochlear nerve in surgical planning for cochlear implants^3^. US is the only one of these modalities available in space flight.

US has distinct advantages over other imaging modalities for potential use to monitor changes to the otolith's size throughout the extended flight. This includes relatively small system size and weight, high resolution, no significant requirements of subject immobilization, and the lack of any ionizing radiation or magnetic fields. Employing effective US monitoring would require the development of a system that would facilitate sufficient sound wave penetration into the inner ear but with adequate resolution to allow effective structure recognition and measurements.

In animal studies, ultrasonic imaging of the external ear canal and middle ear has been used in dogs and cats^4^. The tympanic bulla was visualized in conscious cats by placing the transducer under the chin, medial to the mandible, and aiming vertically^4^. The US was chosen because it was well tolerated in unsedated animals^5^. As noted in the “Discussion” section further, guinea pigs and chinchillas have been used for preclinical research on cochlear implant surgery.

High-frequency imaging of human cadaveric middle ear structures (stapes, incus, and malleus) has been accomplished by individual dissection and scanning^6^. In this study by Brown et al,^6^ the tympanic membrane and a single view of the cochlea were also examined. To our knowledge, no prior study has been reported that demonstrates the detailed sonographic appearance of human inner ear structures. We sought to determine whether pertinent structures of the vestibular system of the inner ear (Fig. 1) could potentially be distinguished with high-frequency US and investigate the best transducer frequencies for image optimization.

## Methods

The relevant areas of 3 human cadaver heads were meticulously dissected to remove overlying bone by the Anatomist coauthor. Care was taken to avoid injury to the middle and inner ear structures. The first author (J.S.) performed all the imaging. He is board-certified in US imaging and a leader in the field of US diagnostic medicine^7^. The anatomic features were identified using conduction gel and Aplio i800 US machine (Canon Medical Systems; https://us.medical.canon/products/ultrasound/transducers/aplio-i-series/). Multiple broadband transducers were utilized to determine the appropriate frequencies for optimal visualization of pertinent anatomy. These included: The Aplio 17 MHz Hockey Stick linear (17LH7), 22 MHz Ultra-high Frequency Hockey Stick linear (i22LH8), Multifrequency Ultra-wideband iDMS Linear (i18LX5), 24 MHz Ultra-high Frequency iDMS Linear (i24LX8), and 33 MHz Ultra-high Frequency iDMS Linear (i33LX9). The Aplio 17 MHz Hockey Stick (17LH7), and 22 MHz Ultra-high Frequency Hockey Stick (i22LH8) are 8 mm small footprint linear transducers (https://us.medical.canon/products/ultrasound/transducers/aplio-i-series/).

The inner ear structures were imaged through the open area of dissection. The middle ear structures were imaged with the transducer placed over the external auditory canal and visualization through the tympanic membrane. Structures to be imaged included 3 semicircular canals, temporal bone, utricle, vestibular nerve, facial nerve, middle ear bones, and otoliths.

## Results

**Figure 2:**
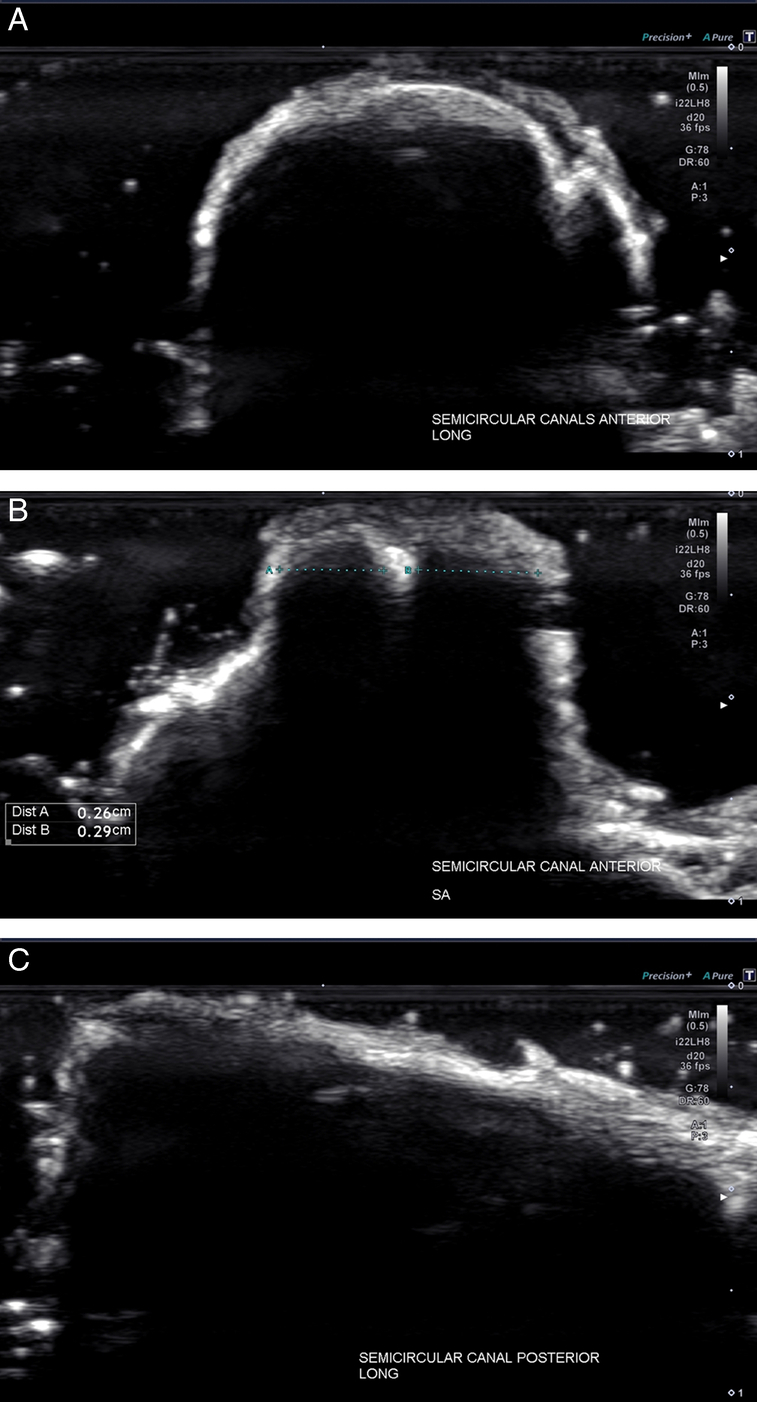
Sonograms showing views of the semicircular canals. The long-axis view of the anterior (A), short-axis view of the lateral and anterior with demonstration of measurement (B), and long-axis view of the posterior (C) are shown. All images were obtained with a 22 MHz small-footprint transducer.

**Figure 3:**
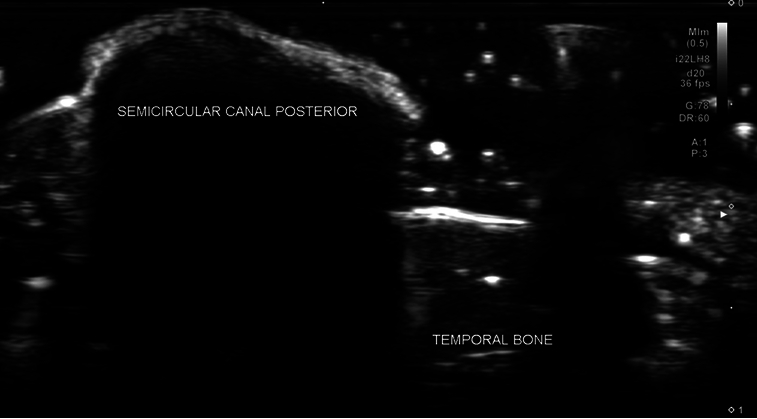
Sonogram (22 MHz) demonstrating differentiation between the echotexture of the semicircular canal and the temporal bone.

**Figure 4:**
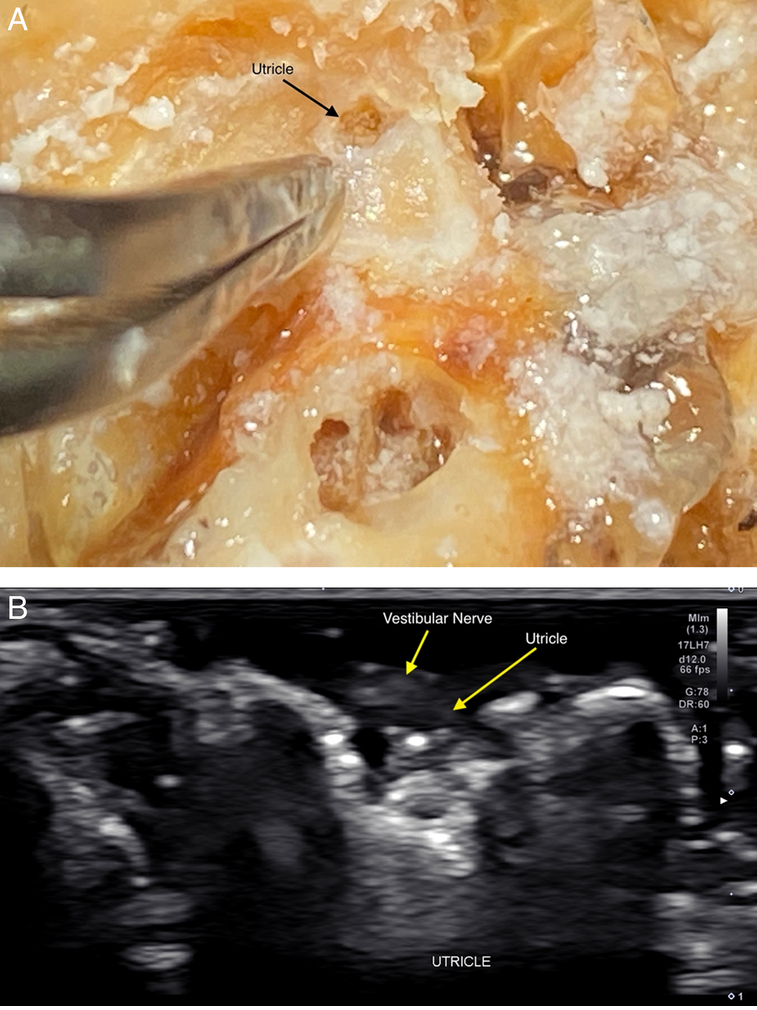
External appearance of the utricle with the vestibular nerve removed (A). Sonogram (17 MHz) demonstrating the identification of the utricle location and overlying short-axis view of a branch of the vestibular nerve (B).

**Figure 5:**
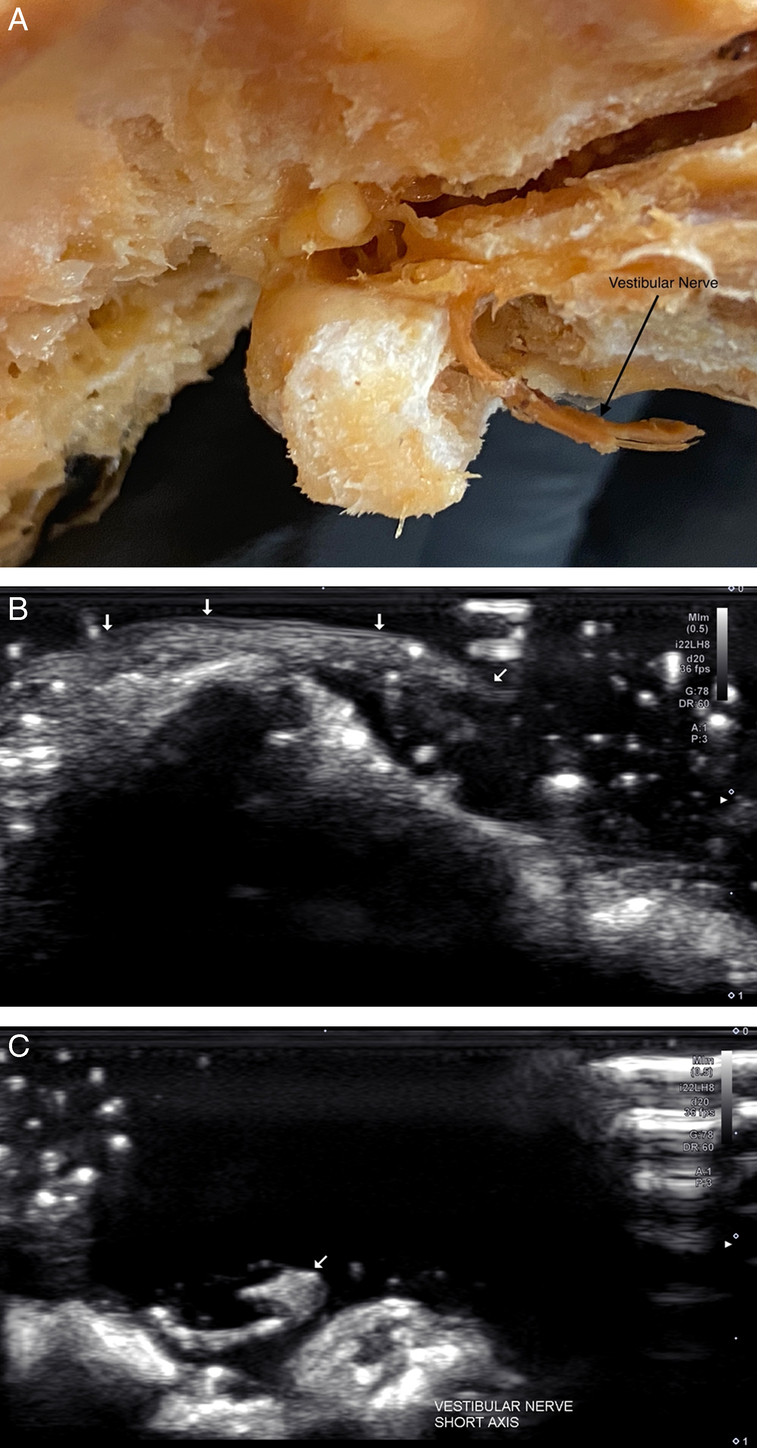
External appearance of the vestibular nerve, that, when identified sonographically, can be followed for localization of the utricle (A). A sonogram (22 MHz) of the vestibular nerve (white arrows) reveals the fascicular architecture in both long-axis (B) and short-axis (white arrow; C).

**Figure 6:**
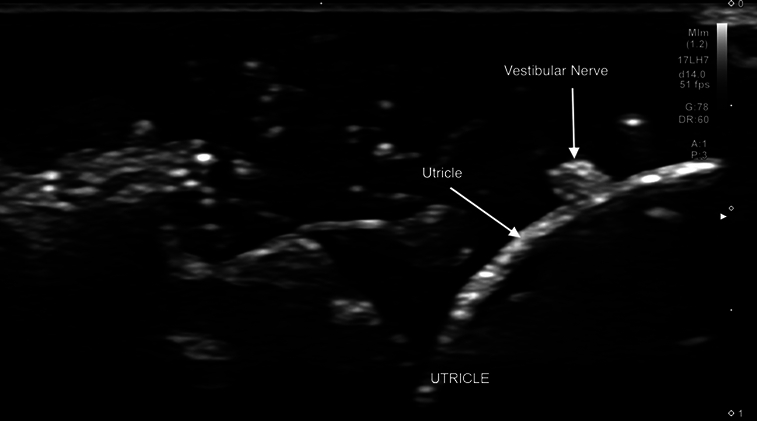
Sonogram (17 MHz) demonstrating the border of the utricle and a short-axis view of the vestibular nerve. The otoliths are deep to the utricle.

**Figure 7:**
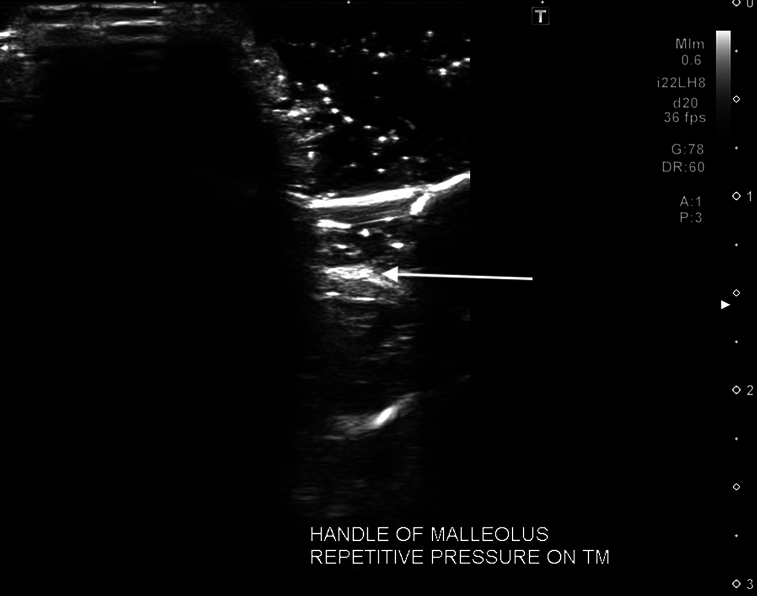
Sonogram (22 MHz) of the middle ear structures including the hammer of the malleus (arrow). The reflection and movement of the middle ear bones were demonstrated with manipulation and dynamic imaging.

**Figure 8:**
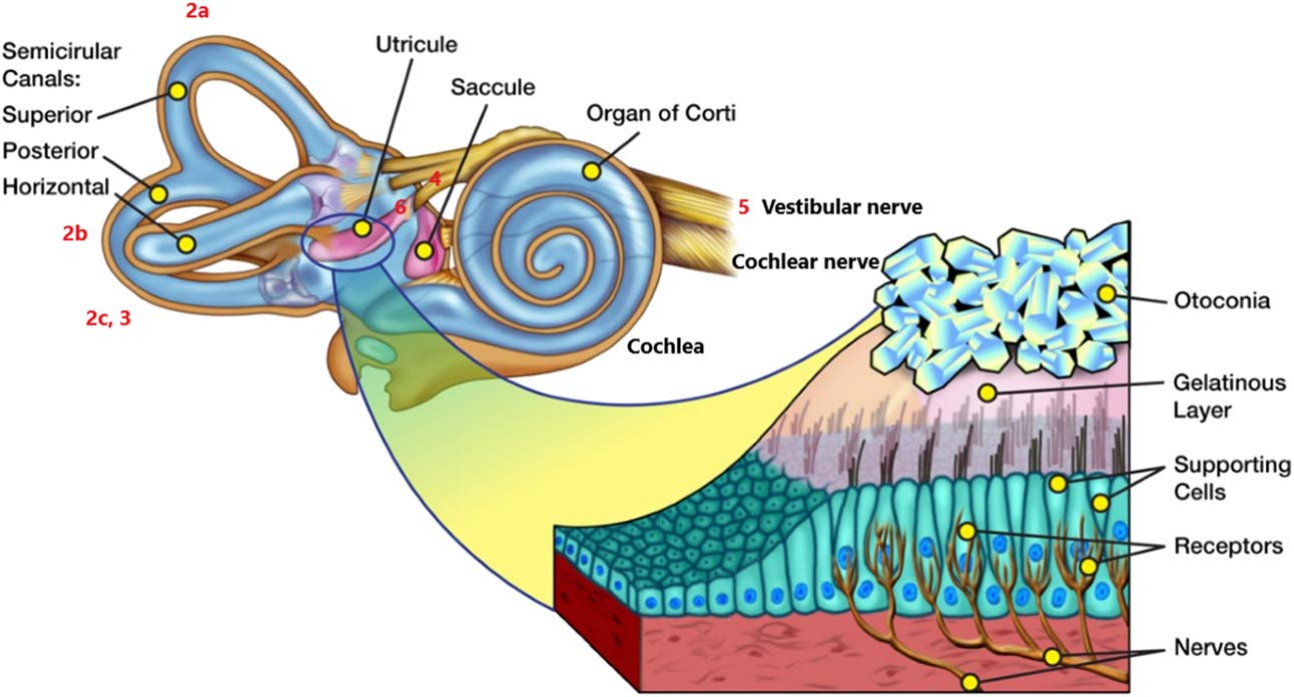
Artwork by Cynthia Bush, NASA (Fig. 1) with the addition of red numbers corresponding to the location of the ultrasound probe placement in Figures 2A–C, 3–6.

In this research setting, it took ~3 hours to complete the survey of ear structures and obtain images that would be ideal for publication presentation. It could potentially take far less time to recognize and evaluate structures in a space traveler with an established protocol. It was determined that the 17 MHz and 22 MHz Hockey stick small footprint linear transducers were most effective for visualization of the desired structures of the inner and middle ear by the ultrasonographer's first author. With respect to the inner ear, it was found that a detailed assessment of all 3 of the semicircular canals (Fig. 2), the cortical surface of the temporal bone (Fig. 3), and the surface of the utricle (Fig. 4), could be performed using multiple transducer directions. Detailed visualization of both the vestibular nerve (Fig. 5) and neighboring facial nerve could be seen, including identification of multiple internal fascicles. In addition, US reflection from the otoliths contained within the utricle was obtained with sufficient clarity to provide a surface measurement (Fig. 6). Bony acoustic landmarks of the middle ear bones were also identified by scanning externally from the tympanic membrane, including dynamic movement of the bones with manual manipulation (Fig. 7). Structures were confirmed by the anatomist and NASA coauthors. The positioning of the probe relative to inner ear structures is illustrated in Figure 8.

## Discussion

### Review of the literature

A review of the literature was performed for imaging of the inner ear. MRI^8–12^ and CT^13,14^ have been used previously. Both T1 and T2 weighted imaging MRIs have captured images of the inner ear^8–10^. 7T MRI has exceeded 3T imaging in recent studies^11,12^. MRI has been used to image the inner ear in diagnosing Meniere disease^15,16^. High-resolution CT has been used to study calcium functional imaging of otoliths^13^ and congenital inner ear anomalies in children^14^. Current imaging systems of this type are not practically feasible for space travel due to limitations of weight and space, as well as the need for containment of ionizing radiation and strong magnetic fields.

There is currently no widely accepted protocol for imaging the inner ear in humans in the US. High-frequency US (HFUS) has been used intraoperatively in guinea pigs to potentially assist in guided cochlear implantation surgery^17^. In this study, the modiolus, scalae vestibule, and tympani were identified to assist with inner ear surgery. HFUS was also used in cadaver cochleas for the purpose of cochlear implantation but required bone decalcification^18^. HFUS combined with micro-CT was also used for cochlear computer-assisted surgery in guinea pigs and found it was possible to visualize cochlear structures^19^. HFUS was also used to vibrate the basilar membrane in chinchilla cochleas^20^.

### High-frequency ultrasound

There is no complete consensus on what defines high-frequency medical US and it varies somewhat with different authors. For our purposes, we are considering conventional medical US frequencies as between 2 MHz and 12 MHz^21^, and high-frequency US is >15 MHz^21^. High-frequency US has the potential to be the ideal imaging system for monitoring human tissue during space travel due to its portability, high spatial resolution, lack of medical or logistical contraindications, and relative ease of image acquisition. Knowledge of imaging details to create successful visualization of the vestibular structures can be used toward the creation of effective imaging systems for the inner ear. If developed, such systems could also potentially be used for other general medical ear imaging applications.

This study demonstrates that many of the pertinent vestibular structures of the inner ear can be adequately identified sonographically with adequate exposure, direction, and penetration of the incident sound waves. This includes the semicircular canals, fascicular details of the vestibular and facial nerve, utricle and reflected otoliths, and bones of the middle ear. Selected middle ear structures including dynamic excursion, can also be seen with imaging through the tympanic membrane. This demonstrates the potential for the development of a US system that could be used to monitor changes within the inner ear.

### Limitations of study

Limitations of the study include visualization in a limited number of cadaver specimens with large, dissected windows for observation and a limited number of observers. It is hoped that further studies with more specimens with potential variation, and narrowing dissected windows, will lead to effective scanning protocols that can allow a sonographer to reliably navigate this complicated anatomic region. With regard to space flight, the weight of machinery is always considered.

## Conclusion

This observational study demonstrates that many of the structures of interest in the inner ear can be adequately visualized and distinguished with the US in cadaveric dissections with adequate exposure. Structures of the middle ear could also be distinguished in the cadaveric specimens by scanning across the tympanic membrane. These findings suggest the potential for developing imaging systems with the US that could monitor changes within the inner ear, including alterations to the vestibular system.

## Ethical approval

None.

## Sources of funding

The authors appreciate the loan of the ultrasound system for research purposes from Canon Medical Systems USA, Inc.

## Author contributions

J.S.. and F.Y.C.T.: concept, design, intellectual content, data acquisition, data analysis, manuscript preparation, editing, and review. H.Z.: design, intellectual content, data acquisition, manuscript editing, and review. M.R.: concept, intellectual content, data analysis, manuscript editing, and review.

## Conflicts of interest disclosures

The authors declare that they have no financial conflict of interest with regard to the content of this report.

## Research registration unique identifying number (UIN)

None.

## Guarantor

None.

## Data Availability

The data that support the findings of this study are available from the corresponding author upon reasonable request.

## Declaration of Generative AI and AI-assisted technologies in the writing process

No AI tools/services were used during the preparation of this work.
